# Relapse of Monoclonal Gammopathy of Renal Significance after mRNA COVID-19 Vaccination: A Case Report

**DOI:** 10.3390/life13030734

**Published:** 2023-03-09

**Authors:** Nikolaos Tsaftaridis, Victoria Potoupni, Lydia Koraka, Fotini Iatridi, Georgios Lioulios, Michalis Christodoulou, Eleni Moysidou, Stamatia Stai, Asimina Fylaktou, Aikaterini Papagianni, Maria Stangou

**Affiliations:** 1 School of Medicine, Aristotle University of Thessaloniki, 54124 Thessaloniki, Greece; 2 Department of Nephrology, Aristotle University of Thessaloniki, Hippokration Hospital, 54642 Thessaloniki, Greece; 3 National Peripheral Histocompatibility Center, Department of Immunology, Hippokration Hospital, 54642 Thessaloniki, Greece

**Keywords:** monoclonal gammopathy of renal significance (MGRS), light chain deposition disease (LCDD), COVID-19 vaccine

## Abstract

This case report represents the first suspected case of light chain deposition disease relapse associated with mRNA COVID-19 vaccination. The 75-year-old female patient of Greek ethnicity was admitted to the clinic for the investigation of worsening renal function detected on routine lab examinations, two weeks after she received the second dose of the Moderna COVID-19 vaccine (mRNA-1273). Rapidly progressive glomerulonephritis and anemia were the most notable findings. She had a history of LCDD, which had remained stable for four years. Serum protein immunofixation showed monoclonal kappa zones, and a bone marrow biopsy revealed 5% plasma cell infiltration. These, along with other investigations, established the diagnosis of LCDD recurrence. The patient was started on chemotherapy, which improved her immunological profile, but not her renal function. The patient has remained on hemodialysis since. The association between mRNA vaccinations and LCDD relapse may be grounds for investigations into the pathophysiology of MGRS, given the patient’s previous long-term remission. This case report is not intended to directly inform changes in clinical practice. We must stress the importance of following all standardized vaccination protocols, especially in immunocompromised patients.

## 1. Introduction

The present case report is, to our knowledge, the first known incident of an adverse effect of the Moderna COVID-19 vaccine (mRNA-1273) in patients with MGRS due to light chain deposition disease.

MGRS is characterized by increased serum levels of a monoclonal immunoglobulin (M-protein) produced by a non-malignant or pre-malignant B cell or plasma cell clone, causing organ damage, and more specifically in our case, renal disease.

As MGRS is associated in its pathophysiology with dysfunctions of B cell maturation and proliferation, there are indications that vaccines targeting these processes, such as mRNA vaccines, may be implicated in exacerbations of the disease [1,2,3]. Furthermore, this patient’s clinical course after vaccination is congruent with this hypothesis, based on the mechanistic understanding proposed by other research results from our clinical center [4].

Since early 2020, severe acute respiratory syndrome coronavirus 2 (SARS-CoV-2), the pathogen behind coronavirus disease 19 (COVID-19), has had a tremendous medical and socioeconomic impact worldwide, and mass vaccination remains a foundational tool for effectively containing its effects on a large scale [5]. Many vaccines have been designed, tested, licensed, and released since the outbreak began and have been proven to be protective, mainly by inducing the activation of memory B cells (MBCs) and long-lived plasma cells (LLPCs). As with any new medical intervention, complications following their administration are sometimes observed, some of them being rare and only in specific populations [6,7].

Describing and understanding the possible side effects of vaccines in general, and each specific vaccine, is important for the improvement of patient care, the further understanding of newer vaccine technologies such as mRNA technology, and for building and retaining trust between patients and healthcare institutions. Furthermore, case reports such as this one can be the basis for explorations into the pathogenesis of LCDD and for the further elucidation of mRNA vaccine mechanisms of action.

## 2. Case Report

A 75-year-old woman of Greek ethnicity presented to the Department of Nephrology of our hospital with rapidly declining renal function. More specifically, microscopic hematuria and notable proteinuria were detected on a routine laboratory examination that she had conducted for clinical monitoring of preexisting disease.

**Figure 1:**
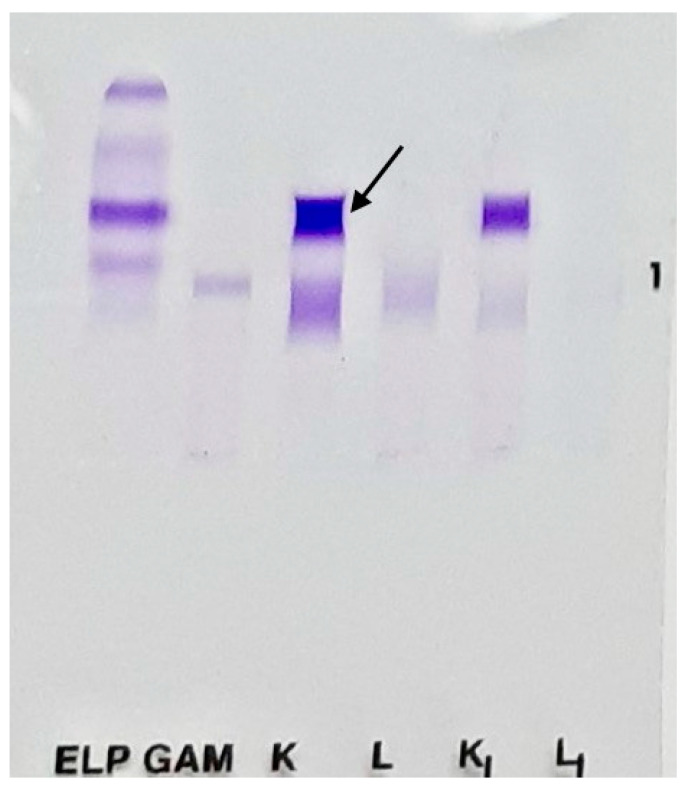
Serum immunofixation showed kappa monoclonal fraction (lanes K and K_I_). Lanes, from left to right: ELP—reference lane, GAM—G/A/M antisera, K—kappa light chain, L—lambda light chain, K_I_—free kappa, and L_I_—free lambda. There was a predominance of kappa light chain (arrow) and free kappa chain (K_I_), which are also visible in the reference lane.

**Figure 2:**
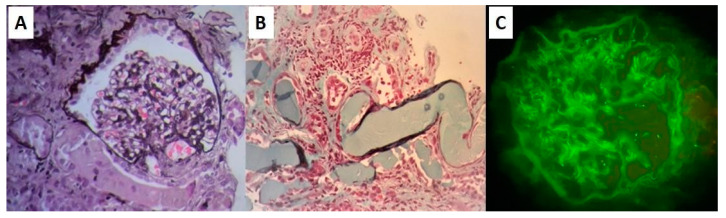
(**A**) A glomerulus showing diffuse proliferation of mesangial cells with increased deposition of mesangial matrix, characteristic of mesangial hyperplasia pattern (periodic acid Schiff–PAS stain). (**B**) Tubules filled with protein deposition (Gomori trichrome (GT)). (**C**) Diffuse mesangial deposition of kappa light chains.

Four years before the time of presentation, at the age of 71 years, she had a similar renal disturbance, which was diagnosed as light chain deposition disease. At the time, serum free light chain levels were elevated, and the kappa to lambda ratio was 22, while both serum and urine immunofixation revealed a monoclonal fraction of kappa light chains, as can be seen in Figure 1. A renal biopsy was taken, and it revealed mesangial proliferation, extended mesangial matrix, tubular atrophy with protein material deposition, interstitial infiltration, and kappa light chain deposits in the glomeruli, as can be seen in Figure 2. A bone marrow biopsy had unremarkable findings, showing only 5% plasma cell infiltration, thus excluding the diagnosis of lymphoma. Immunohistology staining for CD138 in the bone marrow biopsy revealed plasma cells. Unfortunately, cytogenetic studies for this patient were not available to our team.

The patient’s previous medical history also included primary hypertension, hypothyroidism, osteoporosis, and nephrolithiasis, all of which were under proper medical management.

**Figure 3:**
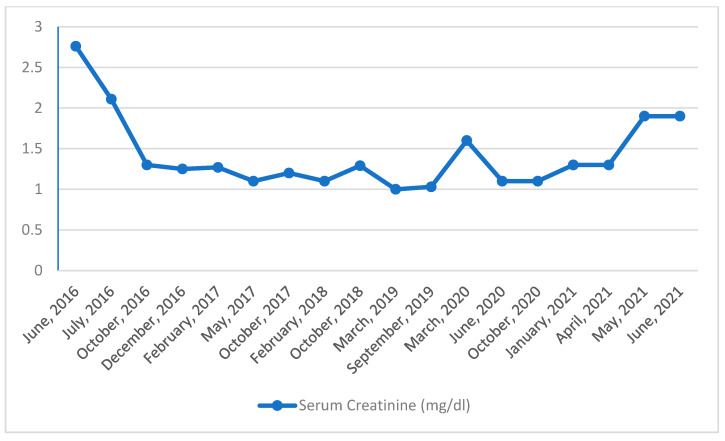
Changes in the patient’s renal function, as reflected by her serum creatinine levels, from the time of the original diagnosis of LCDD until the current relapse. LCDD first presented in 2016 and improved with treatment. The transient increase of serum creatinine in early to mid-2020 reflects an episode of acute renal failure due to diarrheal illness, the cause of which was unrelated to the chronic condition and was treated successfully. Following vaccination against COVID-19 in 2021, her renal function deteriorated and a relapse of her LCDD was diagnosed.

At the time of the initial diagnosis, the patient was started on a corticosteroid regimen, with no additional chemotherapy. She showed a prompt response to the medication, with a rapid reduction in proteinuria, which allowed the treating physicians to avoid any escalations in treatment. Six months later her proteinuria had subsided, κ chains were no longer detectable in serum, and urine samples and her renal function had stabilized, as can be gleaned by her serum creatinine levels, shown in Figure 3. From then on, she entered follow-up on an outpatient basis, with few signs of disease exacerbations for more than 4 years, until 2021.

In that year, two weeks after completing her COVID-19 vaccination with the mRNA-1273 (Moderna) vaccine (two doses separated by a 3-week interval) routine laboratory examinations revealed an acute decline in her renal function. Her serum creatinine levels increased from 1.1 mg/dL (a month previously) to 2.67 mg/dL two weeks after the second dose. In the same period, her EGFR, as calculated using the CKD-EPI equation, declined from 52 to 18 mL/min/1.73 m^2^. Proteinuria also worsened during the same period, from 0.75 g/24 h to 3 g/24 h, and active urine sediment with microscopic hematuria and red blood cell casts were detected in the latter urinalysis specimen.

Patients with circulating M-protein are diagnosed as having monoclonal gammopathy of undetermined significance (MGUS) if the M-protein is <30 g/L. Typically, MGRS exhibits low levels of circulating M-protein, reflecting the small size of the underlying B cell or plasma cell clone [8]. Serum protein electrophoresis (PE) and immunofixation (IF), 24-h urine protein electrophoresis and immunofixation, and a serum free light chain (FLC) assay are included in the recommended diagnostic procedure, whereas a urine FLC assay provides no additional useful information [9,10,11]. International guidelines recommend using a serum FLC assay along with serum protein electrophoresis and immunofixation as an initial screening panel for monoclonal gammopathies [8,12]. Additional genetic tests and fluorescent in situ hybridization studies are helpful for clonal identification and for generating treatment recommendations. Flow cytometry can help identify small clones.

Serum and urine protein electrophoresis and immunofixation, as well as analyses of serum FLC, were performed to identify the monoclonal immunoglobulin, which helped establish the diagnosis of MGRS relapse and could also be useful for assessing the patient’s response to treatment. Finally, bone marrow aspiration and biopsy were conducted to identify the lymphoproliferative clone.

This patient’s immunological profile revealed very high levels of kappa chains, accompanied by monoclonal kappa zones on serum immunofixation and 9% plasma cell infiltration of bone marrow. Immunohistochemistry on the bone marrow biopsy also showed a predominance of CD138+ cells. These findings supported the diagnosis of recurrent LCDD and secondary renal involvement.

In general, the chemotherapeutic agents used to treat MGRS are those that target plasma cells or other B cell neoplasms. Such agents include proteasome inhibitors (e.g., bortezomib, carfilzomib, and ixazomib), monoclonal antibodies (e.g., rituximab and daratumumab), alkylating agents (e.g., cyclophosphamide, bendamustine, and melphalan), immunomodulatory drugs (e.g., thalidomide, lenalidomide, and pomalidomide), and glucocorticoids (e.g., prednisone and dexamethasone) or human immunoglobulin replacement therapy [13]. In some patient populations, such as those with amyloidosis or monoclonal immunoglobulin deposition disease, the treatment strategy may also involve autologous hematopoietic cell transplantation and chemotherapeutic agents that do not require dose modification for kidney function.

In our case, the patient was commenced on the standard treatment protocol, with bortezomib, cyclophosphamide, and dexamethasone. The therapeutic approach was organized around 21-day treatment cycles, with:

- Intravenous cyclo-phosphamide 500 mg on the first day of each treatment cycle.
- Intravenous bortezomib 1.3 mg/m^2^ on days 1, 4, 8 and 11, followed by a 10-day hiatus.
- Oral dexamethasone 20 mg, on days 1, 2, 4, 5, 8, 9, 11, 12.

She received only two treatment cycles; then, given the decline in her renal function, she was started on hemodialysis. Therefore, bortezomib doses had to be reduced to 0.7 mg/m^2^ on the treatment schedule outlined above, and administered after her dialysis sessions.

Despite treatment, her renal function deteriorated, and she entered hemodialysis. Four months later, her immunological profile had improved, with undetectable serum free light chains, but her renal function did not improve, and the patient remained on regular hemodialysis.

The decline in renal function despite the improvement in the patient’s immunological status was attributed to the aggravation of chronic renal lesions. Chronic renal involvement, including membranoproliferative lesions and tubulointerstitial inflammation, cannot be completely reversed by treatment; secondary focal segmental sclerosis and tubular atrophy are inevitable, as has been proven in other progressive glomerular diseases. Moreover, our patient had a mild baseline renal impairment with an episode of acute renal failure during the four-year follow-up, which was strongly indicative of chronic sclerotic lesions.

## 3. Discussion

The definition of MGRS was initially described in 2012 by the International Kidney and Monoclonal Gammopathy Research Group (IKMG) and later refined in 2017, when the diagnostic criteria for MGRS-related diseases were updated [1,2,3].

Monoclonal gammopathies (MGs) are directly associated with the dysregulation of B cell maturation and proliferation processes, as the responsible monoclonal immunoglobulin is produced in excess by an indolent B cell clone. Monoclonal gammopathy of undetermined significance (MGUS) is a disease entity defined by the presence of paraprotein and hematologic findings in line with, but unsatisfactory for the diagnosis of, multiple myeloma [14]. More specifically, MGUS is diagnosed with <10% bone marrow plasma cell representation and a lack of B cell aggregates [14].

MGRS is a term introduced to highlight that MGUS is not a benign disease that should only be monitored, as previously thought [1]. It highlights the renal damage caused by monoclonal gammopathy of undetermined significance, a disorder hematologically consistent with MGRS, but apparently without end-organ involvement [14].

Interestingly, MGRS was recently recognized and formally defined in the “5th Edition of the World Health Organization of Haematolyphoproliferative Tumours: Lymphoid Neoplasms” as follows: “Monoclonal gammopathy of renal significance (MGRS) represents a plasma cell or B-cell proliferation that does not meet accepted criteria for malignancy but secretes a monoclonal immunoglobulin or immunoglobulin fragment resulting in kidney injury” [15].

The diagnosis of MGRS affecting the kidneys is established via renal biopsy in cases of high clinical suspicion. Characteristic findings in optical microscopy and immunofluorescence include the membranoproliferative or mesangial hyperplasia pattern of glomerulopathy, followed by monotypic immunoglobulin and complement deposition.

Secondary causes of MGRS may include systemic diseases, malignancies, viral or bacterial infections, and overall, any factor that stimulates the proliferation of B cells [16]. Vaccines may be included in these factors, as B cell proliferation is their main target of action.

Renal biopsy registries show that a diagnosis of light chain deposition disease (LCDD) is made in 0.3–0.5% of all kidney biopsies, with an identified underlying monoclonal gammopathy of undetermined significance in approximately 41% of these cases [17,18]. All other types of MGRS have unknown incidence and/or prevalence.

Kidney lesions in MGRS are primarily caused by the abnormal deposition of monoclonal proteins. Monoclonal proteins produced may be light chains, heavy chains, or intact whole immunoglobulins; they are produced by small, nonmalignant, or premalignant plasma cell or B cell clones. Deposition of these proteins may occur within the glomeruli, tubules, vessels, or the interstitium, depending upon the specific biochemical characteristics of the pathogenic light and/or heavy chains involved.

The deposits can be categorized as organized or nonorganized. MGRS lesions with organized deposits can be further subdivided into those with fibrillar deposits, microtubular deposits, or crystal inclusions. MGRS lesions with nonorganized deposits include the monoclonal immunoglobulin deposition diseases (MIDDs; light chain, heavy chain, or light and heavy chain deposition diseases) and monoclonal gammopathy-associated proliferative glomerulonephritis, involving monoclonal immunoglobulin G (IgG), and rarely immunoglobulin A (IgA), immunoglobulin M (IgM), or light chain-only deposits. MGRS lesions without deposits include thrombotic microangiopathy associated with monoclonal gammopathy [19].

Other mechanisms regarding the pathogenesis of MGRS have also been described:

1. Secretion of high levels of vascular endothelial growth factor [20,21,22,23].
2. Monoclonal proteins can act as autoantibodies directed against complement components.
3. Circulating monoclonal immunoglobulin autoantibodies can target the phospholipase A2 receptor and induce a form of membranous nephropathy that can rapidly recur after kidney transplantation [24,25].
4. Light chain (AL), heavy chain (AH), and heavy and light chain (AHL) amyloidosis. Extracellular deposition of amyloid in glomeruli, tubules, and/or vessels is characteristic of renal amyloidosis. In most cases, the M-protein-related amyloidosis is derived from fragments of monoclonal light chains (LCs), which are more often of the lambda (λ) than kappa (κ) isotype 26, and rarely from fragments of intact immunoglobulin (Ig) or heavy chains only. Amyloid is the only MGRS lesion that is Congo red-positive [8].

Kidney biopsy is crucial not only to establish a diagnosis of MGRS and differentiate it from MGUS, but also to describe the exact type of renal pathology.

Patients with MGRS usually present with a progressive decline in kidney function, microscopic hematuria, proteinuria ranging from sub-nephrotic to overt nephrotic syndrome, electrolyte abnormalities, and/or proximal tubular dysfunction [8]. Although MGRS is commonly seen in patients 50 years old or older, it has also been reported in younger patients.

Several studies of patients with MGRS have shown that kidney outcomes are closely associated with the hematologic response to chemotherapy [8,26,27,28,29]. The treatment approach should be directed against the pathologic clone, with the primary goal of preserving kidney function.

Regarding the recurrence of lymphoproliferative disorders, several environmental factors may be involved, such as bacterial infections and medications. Patients with MGRS are routinely vaccinated against seasonal influenza, H1N1, and Streptococcus pneumoniae before treatment initiation. Thus, this may be the main reason why there are no data regarding disease relapse following vaccination.

Vaccination against COVID-19 aims to provoke an adequate cellular and humoral immune response, which includes a shift of B lymphocytes to specific subtypes capable of producing antibodies and establishing immune memory [30]. Recent studies have shown that levels of CD4+CD38+HLADR+ and CD8+CD38+HLADR+ activated T cells are substantially increased three weeks after vaccination [30]. Our results also showed that antibodies against the receptor binding domain of SARS-Cov-2 S protein, as well as neutralizing antibodies, were raised in CKD patients after mRNA vaccination for COVID-19, with the main increase at the first and second month after the second dose [4]. This timeframe corresponds to the time our patient had a relapse.

Similar effects from other vaccines regularly administered to these patients may be attenuated by routine vaccination before the initiation of treatment, thus hiding the effect. Vaccines in this category include those against seasonal influenza, H1N1, and Streptococcus pneumoniae.

## 4. Conclusions

Our case may be an example of the potential pathogenic effects of immune stimulation after vaccination in patients with monoclonal gammopathy of renal significance. Laboratory findings pointed to a relapse of the underlying chronic disease with an aggravation of chronic kidney lesions, as revealed on biopsy. The previous long-term remission the patient was in, along with the timing of the LCDD flare-up, are signs of a possible underlying association. Despite adequate medical treatment, the patient had to enter dialysis and has remained on it ever since.

The introduction of new vaccines and vaccine technologies, such as the mRNA vaccine, may be a good opportunity to study plasma cell dysfunction, such as in this disease. The mechanism of action of vaccines, in that they stimulate B cells to proliferate and differentiate and produce immunoglobulins, may affect the balance sought after in patients with monoclonal gammopathies. It is possible that if more cases such as this one are noted, changes or modifications may be explored toward the improvement of patient outcomes regarding both vaccination and the management of MGRS, as well as monoclonal gammopathy of clinical significance more generally.

Along with the above hypotheses, we must stress again that we urge clinicians and patients to properly follow standardized routine mandates regarding Sars-CoV-2 vaccination, especially in immunocompromised patients. This clinical case report and brief review of the literature regarding MGRS does not entail reliable data capable of informing changes in clinical practice. This case report may simply point to a need for more research and clinical vigilance in this area of practice, as well as an opportunity to find out whether there are any associations between mRNA vaccination and LCDD relapse, and, if so, to ascertain the underlying pathophysiology and sequelae for clinical practice.
